# Thermoelectric Effect in a Correlated Quantum Dot Side-Coupled to Majorana Bound States

**DOI:** 10.1186/s11671-020-03307-y

**Published:** 2020-04-15

**Authors:** Feng Chi, Zhen-Guo Fu, Jia Liu, Ke-Man Li, Zhigang Wang, Ping Zhang

**Affiliations:** 1 School of Electronic and Information Engineering, University of Electronic Science and Technology of China, Zhongshan Institute, Shiqi District Xueyuan Road No. 1, Zhongshan, 528402 China; 2 Institute of Applied Physics and Computational Mathematics, No. 6 Huayuan Road, Haidian District, Beijing, 100088 China; 3 School of Science, Inner Mongolia University of Science and Technology, Kundu District Alding Road No. 7, Baotou, 014010 China

**Keywords:** Thermoelectric effect, Quantum dot, Majorana bound states, Thermopower

## Abstract

We theoretically study the thermoelectric effect in a hybrid device composed by a topological semiconducting nanowire hosting Majorana bound states (MBSs) and a quantum dot (QD) connected to the left and right non-magnetic electrodes held at different temperatures. The electron-electron Coulomb interactions in the QD are taken into account by the non-equilibrium Green’s function technique. We find that the sign change of the thermopower, which is useful for detecting the MBSs, will occur by changing the QD-MBS hybridization strength, the direct overlap between the MBSs at the opposite ends of the nanowire, and the system temperature. Large value of 100% spin-polarized or pure spin thermopower emerges even in the absence of Zeeman splitting in the QD or magnetic electrodes because the MBSs are coupled to electrons of only one certain spin direction in the QD due to the chiral nature of the Majorana fermions. Moreover, the magnitude of the thermopower will be obviously enhanced by the existence of MBSs.

## Introduction

The preparation and detection of zero-energy Majorana bound states (MBSs) are of particular importance in modern condensed matter physics. Fundamentally, the MBSs are solid state counterpart of Majorana fermions and are associated with non-Abelian statistics that can enable topologically protected quantum information with potential applications in quantum computation free from decoherence [1–3]. Apart from this, the MBSs are also promising in design of high-efficiency electronic devices, such as the spintronics [4]. Well-separated MBSs can be prepared in various systems, of which the most important schemes include non-centrosymmetric superconductors [5], three- or two-dimensional topological insulators coupled to superconductors [6], electrostatic defects in topological superconductors [7], p-wave superconductors [8], the semiconducting [9] or ferromagnetic [10] nanowires with native strong spin-orbit interaction proximitization to a conventional s-wave superconductors, and Josephson junctions [11].

As for the detection of MBSs, it is also quite challenging because the Majorana fermions are their own antiparticles and charge-neutral due to their intrinsic particle-hole symmetry. A variety of experiments have been carried out to verify the existence of MBSs through phenomena such as the 4*π* periodic Josephson current phase in junctions between topological superconductors [12], half-integer conductance plateau at the coercive field in a hybrid structure composing of topological superconductors and topological quantum anomalous Hall insulator [13], tunneling spectroscopy using Rashba nanowires coupled to the bulk s-wave superconductors [14], and zero bias of the differential conductance at the edges of the wires [14, 15]. However, these phenomena have other possible physical origins except for MBSs, and alternative schemes then have been proposed. One of them is the hybridization of MBSs with other nanoscale structures, such as the zero-dimensional quantum dot (QD) in which the energy levels, electron-electron Coulomb interactions, particle numbers, and coupling strength to external environment are all well controllable [16, 17]. At low temperature, a half-maximum conductance when the energy level of the QD is aligned to the Fermi energy in the leads was theoretically predicted as a clear evidence of the formation of a pair of MBSs [18]. This result is completed unchanged by the adjusting of the QD energy level [19] and has successfully been observed in experiment in a QD coupled to an InAs-Al nanowire [20]. Recently, optical schemes based on QD structure were also theoretically proposed to detect the MBSs with the help of optical pump-probe technique. [21, 22] In ring- or T-shaped QD-based systems, the quantum interference phenomena are drastically affected by the MBSs [23–25] and then can be used for the detection scheme with the help of, for example, the Fano effect [26–28].

Recently, there are also some work concerning detection of the MBSs via thermoelectric effect, which focuses on the conversion between electrical and thermal energies. This old research topic gains renewed attention due to the rapid progress of growth and fabrication of mesoscopic devices and nanostructures, in which the thermoelectric performances are obviously improved [29, 30]. High-efficiency energy harvesters based on QDs that are defined on such as a GaAs/AlGaAs interface two-dimensional electron gas have recently been reported [31, 32]. Enhancement of the thermoelectric effect in them can be attributed to the considerable reduction of the thermal conductivity by boundary scattering and the optimization of the electrical transport properties unique in these low-dimensional systems [30–32]. The thermopower (Seebeck coefficient) is the central quantity in thermoelectric effect. It is the strength of a open-circuit voltage in response of a temperature gradient applied in a solid material with free electronic carriers. Hou et al. theoretically predicted that the thermopower between a QD and superconductor hosting a Majorana edge state satisfies the Mott formula and generically does not vanish by using the Landauer-Büttiker formalism [33]. Based on such a property, one can infer the temperature of the Majorana edge state by measuring the differential conductance and the thermopower. Leijnse demonstrated theoretically that the coupling between a QD with tunable energy level and MBSs breaks particle-hole symmetry, and the changes of thermopower provide a new way of proving the existence of Majorana states [34]. The thermoelectric properties in such a setup can also be used to detect the temperature of the superconductor and to extract information about the dissipative decay of MBSs [34]. In a structure with a QD coupled to two electrodes, López et al. showed that the thermopower will change its sign by changing the direct hybridization between the MBSs, a good evidence of the existence of MBSs [35]. The sign change of the thermopower was also subsequently found in systems of a QD with two [36] or three [37] electrodes. Moreover, it was demonstrated that the relationship between the shot noise and thermoelectric quantities may provide a purely electrical way to detect the charge-neutral MBSs [38, 39].

**Fig. 1:**
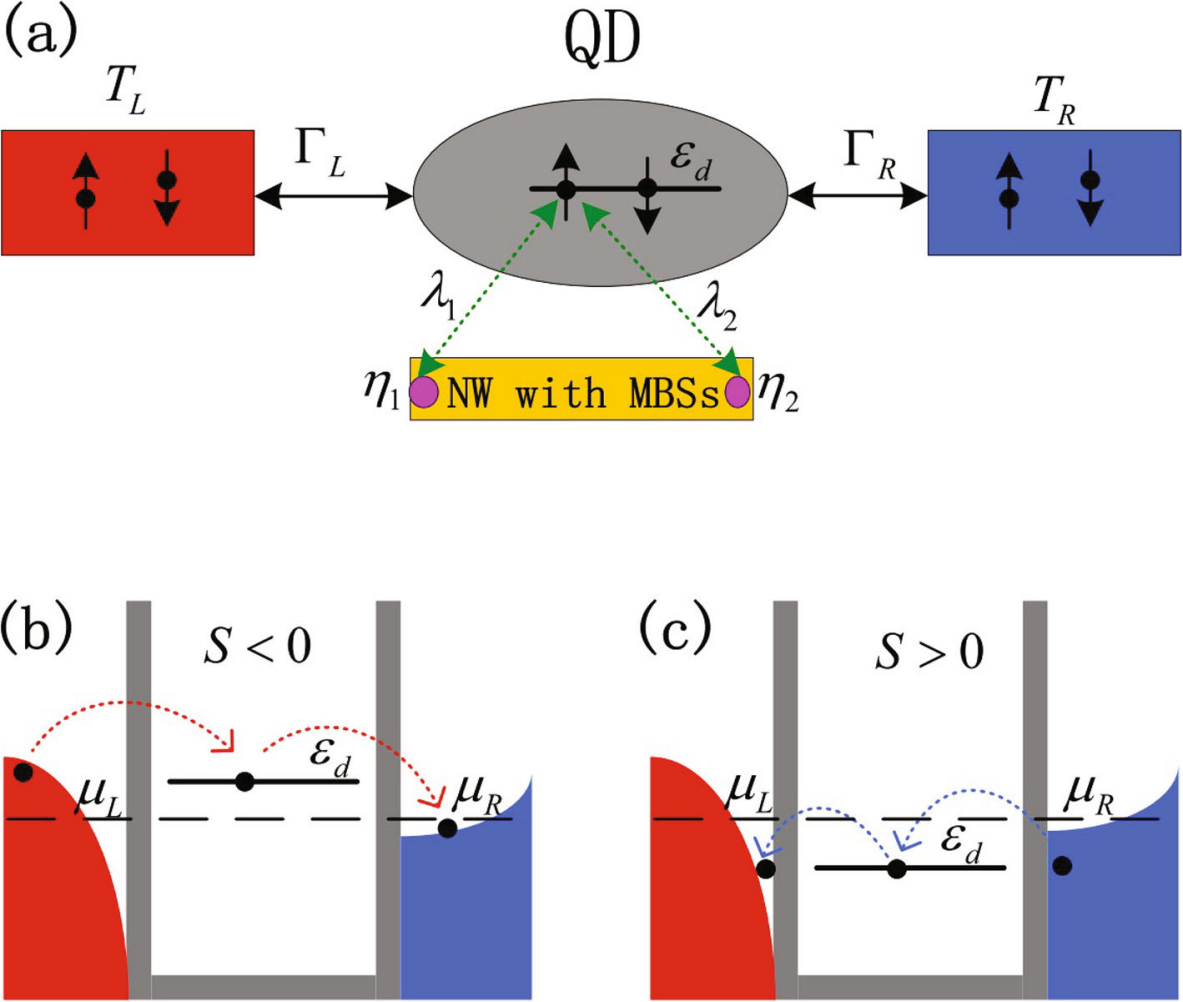
Schematic of the model (color online). **a** Schematic of the simulation structure composed by a QD with gate-tunable energy level *ε*_*d*_ which can be occupied by either a spin-up or a spin-down electron. The QD is connected to the left and right leads held at different temperatures with coupling strength *Γ*_*L*/*R*_. The MBSs *η*_1/2_ are formed in the ends of the semiconducting nanowire and are coupled to the spin-up electrons in the QD due to the chiral nature of the Majorana fermions with strengths of *λ*_1_ and *λ*_2_, respectively. The energy state of spin-up electrons will be changed by MBSs-QD coupling, and then, the strength and sign of the thermopower *S* will be influenced. In the present model, we assume the temperature of the left lead *T*_*L*_ is higher than that of the right one *T*_*R*_, and then, there are more electrons (empty states) being excited above (below) the chemical potential in the left lead than those in the right lead. **b**, **c** The electron tunneling processes and the resulted thermopower in the absence of MBSs-QD coupling. In **b**, the QD energy level *ε*_*d*_ is above the chemical potential of the leads *μ*_*L*/*R*_=*μ*, and then, electrons from the occupied states *ε*_*d*_>*μ* in the left hotter lead will tunnel through dot state *ε*_*d*_ to the empty state in the right colder lead, resulting in negative thermopopwer *S*<0. In **c**, *ε*_*d*_<*μ*, and then, the sign of the thermopower is reversed accordingly

In the present paper, we propose a hybridized system composing of MBSs and a QD coupled to electrodes (see Fig. 1) to study the properties of the thermopower. In the nanosystem we considered, the strong Coulomb interaction in the dot, which has been neglected in previous works [18, 22–24, 34–39], is taken into account. Furthermore, we consider that only one spin component of the QD spin is coupled to the MBSs due to the chiral nature of the MBSs [40]. We find that the sign of the thermopower can be effectively reversed by changing the dot-MBSs coupling strength, the direct hybridization between the MBSs, and the system temperature. The resulted large 100% spin-polarized and pure spin thermopower, which are the corresponding 100% spin-polarized and pure spin currents in closed circuit, are useful in spintronics. The coupling of both the two MBSs to the QD will further enhance the magnitude of the thermopower, but does not change the essential results when only one of the MBSs is coupled to the dot. Based on the presently advanced quantum transport measurements for the MBSs through QD coupled with topological superconducting nanowires, we believe our proposal could be experimentally tested in the future. Additionally, our proposal and findings in this work may provide an excellent way to detecting the formation of the MBSs in QD.

## Model and Methods

The effective Hamiltonian of the QD coupled to MBSs and the left and right normal metal electrodes takes the following form [34, 35]:

1 $$\begin{array}{@{}rcl@{}} H & =\sum_{k\beta\sigma}\varepsilon_{k\beta}c_{k\beta\sigma}^{\dag}c_{k\beta\sigma} +\sum_{\sigma}\varepsilon_{d}d_{\sigma}^{\dag}d_{\sigma}+Ud_{\uparrow}^{\dag} d_{\uparrow} d_{\downarrow}^{\dag} d_{\downarrow} \\ & +\sum_{k\beta\sigma}(V_{k\beta}c_{k\beta\sigma}^{\dag}d_{\sigma}+H.c)+H_{\text{MBSs}}, \end{array} $$

where $c_{k\beta \sigma }^{\dag } (c_{k\beta \sigma })$ creates (annihilates) an electron of momentum *k*, energy *ε*_*kβ*_ (its dependence on spin is neglected for normal metal electrode), and spin *σ*=*↑*,*↓* in electrode *β*=*L*,*R*. For the QD, $d_{\sigma }^{\dag } (d_{\sigma })$ is the creation (annihilation) operator of an electron with gate voltage tunable energy level *ε*_*d*_, spin- *σ*, and intradot Coulomb interaction *U*. The coupling strength between the QD and the leads is described by *V*_*kβ*_. The last term *H*_MBSs_ in Eq. (1) stands for the zero-energy MBSs located on opposite ends of the semiconducting nanowire and their coupling to the QD [18]:

2 $$\begin{array}{@{}rcl@{}} {}H_{\text{MBSs}}=i\delta_{M}\eta_{1}\eta_{2}+\lambda_{1}(d_{\uparrow}-d_{\uparrow}^{\dag})\eta_{1}+i\lambda_{2}(d_{\uparrow}+d_{\uparrow}^{\dag})\eta_{2}, \end{array} $$

in which *δ*_*M*_ is the overlap amplitude between the two MBSs with operator satisfying both $\eta _{j}=\eta _{j}^{\dag } (j=1,2)$ and {*η*_*i*_,*η*_*j*_}=*δ*_*i*,*j*_. The hopping amplitude between MBSs and spin- *↑* electrons in the QD is accounted by *λ*_*j*_. It is helpful to write *η*_*j*_ in terms of the regular fermionic operators *f* as [18] $\eta _{1}=(f^{\dag }+f)/\sqrt {2}$ and $\eta _{2}=i(f^{\dag }-f)/\sqrt {2}$, and then, *H*_MBSs_ is rewritten as:

3 $$\begin{array}{*{20}l} H_{\text{MBSs}}&=\delta_{M}\left(f^{\dag} f-\frac{1}{2}\right)+\frac{\lambda_{1}}{\sqrt{2}}\left(d_{\uparrow}-d_{\uparrow}^{\dag}\right)\left(f^{\dag} +f\right)\\&-\frac{\lambda_{2}}{\sqrt{2}}(d_{\uparrow}+d_{\uparrow}^{\dag})\left(f^{\dag}-f\right). \end{array} $$

We consider the system in linear response regime, i.e., under infinitely small bias voltage *ΔV* and temperature difference *ΔT* between the left and right leads, the electric and heat currents of each spin component are obtained as:

4 $$\begin{array}{*{20}l} &I_{e,\sigma}=-e^{2}L_{0,\sigma}\Delta V+\frac{e}{T}L_{1,\sigma}\Delta T, \end{array} $$

5 $$\begin{array}{*{20}l} &I_{h,\sigma}=eI_{1,\sigma}\Delta V-\frac{1}{T}L_{2,\sigma}\Delta T, \end{array} $$

where *e* is the electron charge and *T* the system equilibrium temperature, and

6 $$\begin{array}{@{}rcl@{}} L_{n,\sigma}=\frac{1}{\hbar}\int (\varepsilon-\mu)^{n}\left[-\frac{\partial f(\varepsilon,\mu)}{\partial \varepsilon}\right]T_{\sigma}(\varepsilon)\frac{d\varepsilon}{2\pi}, \end{array} $$

where $\hbar $ is the reduced Planck’s constant. We set the leads’ chemical potential *μ*=0 as the energy zero point. The Fermi distribution function is given by *f*(*ε*,*μ*)=1/{1+exp[(*ε*−*μ*)/*k*_*B*_*T*]} with *k*_*B*_ being the Boltzmann constant. The transmission coefficient *T*_*σ*_(*ε*) is calculated with the help of the retarded Green’s function as:

7 $$\begin{array}{@{}rcl@{}} T_{\sigma}(\varepsilon)=\frac{\Gamma_{L}\Gamma_{R}}{\Gamma_{L}+\Gamma_{R}} [-2\text{Im}G_{\sigma}^{r}(\varepsilon)], \end{array} $$

where $\Gamma _{L(R)}=2\pi \sum _{k}|V_{kL(R)}|^{2}\delta [\varepsilon -\varepsilon _{kL(R)}]$ is the line-width function. We apply the standard equation of motion technique to obtain Green’s function. The higher-order Green’s functions are truncated by following scheme 2 in ref. [39], i.e., neglect the simultaneous tunneling of the electron of opposite spin. After some straightforward calculations, the spin-up retarded Green’s function is given by:

8 $$ {\begin{aligned} G_{\uparrow}^{r}(\varepsilon)=\frac{\varepsilon_{-}-\Sigma^{M}_{1}-U\left\{1-< n_{\downarrow}>\left[1-(\lambda_{1}^{2}-\lambda_{2}^{2})^{2}\tilde{B}\tilde{B}_{U}\right]\right\}}{\left(\varepsilon_{-}-\Sigma^{M}_{0}\right)\left(\varepsilon_{-}-U-\Sigma^{M}_{1}\right)}, \end{aligned}}  $$

where the MBS-induced self-energies

9 $$ \Sigma^{M}_{0}=B_{1}+\left(\lambda_{1}^{2}-\lambda_{2}^{2}\right)^{2}B\tilde{B},  $$

and

10 $$ \Sigma^{M}_{1}=B_{1}+\left(\lambda_{1}^{2}-\lambda_{2}^{2}\right)^{2}B\tilde{B}_{U},  $$

with

11 $$\begin{array}{*{20}l} &B=\frac{\varepsilon}{\varepsilon^{2}-\delta_{M}^{2}}, \end{array} $$

12 $$\begin{array}{*{20}l} &B_{1}=\frac{1}{2}\left(\frac{\lambda_{1}^{2}-\lambda_{2}^{2}}{\varepsilon-\delta_{M}}+\frac{\lambda_{1}^{2}+\lambda_{2}^{2}}{\varepsilon+\delta_{M}}\right), \end{array} $$

13 $$\begin{array}{*{20}l} &\tilde{B}=\frac{B}{\varepsilon_{+}+B_{2}}, \end{array} $$

14 $$\begin{array}{*{20}l} &\tilde{B}_{U}=\frac{B}{\varepsilon_{+}+U-B_{2}}, \end{array} $$

in which

15 $$ B_{2}=\frac{1}{2}\left(\frac{\lambda_{1}^{2}-\lambda_{2}^{2}}{\varepsilon+\delta_{M}}+\frac{\lambda_{1}^{2}+\lambda_{2}^{2}}{\varepsilon-\delta_{M}}\right),  $$

and *ε*_±_=*ε*±*ε*_*d*_+*i*(*Γ*_*L*_+*Γ*_*R*_)/2. In the absence of dot-MBSs hybridization (*λ*_1_=*λ*_2_=0), we have $\Sigma ^{M}_{0,1}=0$ and $G_{\uparrow }^{r}(\varepsilon)$ recovers that of ref. [39]. It is also the spin-down retarded Green’s function by changing *n*_*↓*_ into *n*_*↑*_. The occupation number is calculated self-consistently from:

16 $$\begin{array}{@{}rcl@{}} n_{\sigma}=\int \frac{d\varepsilon}{2\pi}\frac{\Gamma_{L}f_{L}(\varepsilon)+\Gamma_{R}f_{R}(\varepsilon)}{\Gamma_{L}+\Gamma_{R}}[-2\text{Im}G_{\sigma}^{r}(\varepsilon)], \end{array} $$

where *f*_*L*/*R*_(*ε*) is the Fermi distribution function in the left/right electrode.

Once the transmission function is obtained from Green’s function, the electrical conductance and the thermopower (Seebeck coefficient) of each spin component are given by *G*_*σ*_=*e*^2^*L*_0,*σ*_ and *S*_*σ*_=−*L*_1,*σ*_/(*eTL*_0,*σ*_), respectively.

## Results and Discussions

**Fig. 2:**
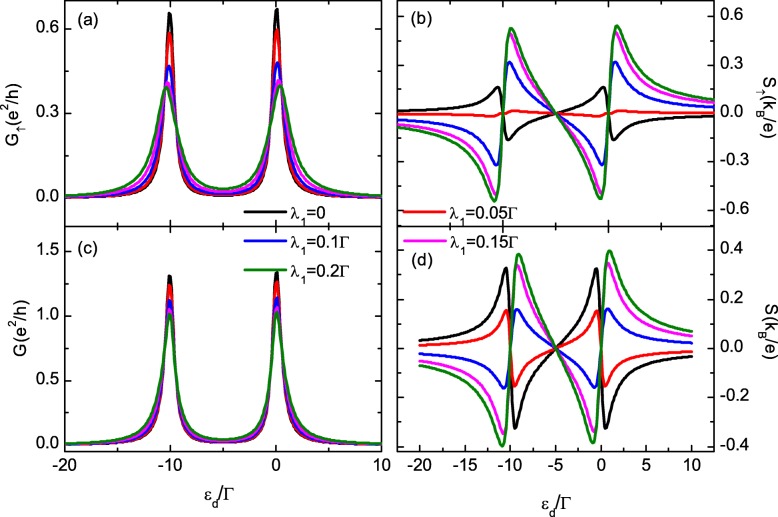
Spin-dependent conductance and thermopower for different dot-Majorana coupling strengths (color online). The spin-up and total conductance in **a**, **c** and thermopower in **b**, **d** verse dot-level. The spin-down conductance and thermopower are almost unchanged by the dot-Majorana coupling strength *λ*_1_, and they overlap with the black solid lines in **a** and **c**, respectively. Other parameters are temperature *T*=0.025*Γ*,*Δ*_*M*_=0,*U*=10*Γ*, and *λ*_2_=0

In what follows, we assume symmetric coupling between the QD and electrodes, and set *Γ*=2*Γ*_*L*_=2*Γ*_*R*_=1 as the energy unit. The intradot Coulomb interaction is fixed as *U*=10*Γ*. We first study the case of the QD which is coupled to only MBS-1 with different hybridization strength *λ*_1_ in Fig. 2 by setting *λ*_2_=0. For *λ*_1_=0, the conductance of each spin component in Fig. 2a develops two peaks located respectively at *ε*_*d*_=−*μ* and −*μ*−*U*. Note now the QD is free from spin polarization induced by the MBS, and the conductance of the two spin component is equal to each other (*G*_*↑*_=*G*_*↓*_), accordingly. Turning on the hybridization between the MBS and the QD (*λ*_1_≠0), the magnitude of *G*_*↑*_ is monotonously suppressed as shown in Fig. 2a, which is consistent with previous results [18, 34, 35]. The value of *G*_*↓*_, however, is almost unchanged even the occupation number *n*_*↓*_ is changed by *λ*_1_ due to the presence of intradot Coulomb interaction (which is not shown in the figure). Meanwhile, the peaks’ position and width in *G*_*↑*_ are slightly modified by the value of *λ*_1_ due to the level renormalization by the dot-Majorana coupling [18, 34, 35]. The configuration of the total conductance *G*=*G*_*↑*_+*G*_*↓*_ in Fig. 2c resembles that of *G*_*↑*_.

The thermopower *S*_*↑*_ in Fig. 2b shows the typical sawtooth configuration and has three zero points individually at *ε*_*d*_=*μ*,−*U*/2, and *μ*−*U* [41, 42]. It develops a pair of sharp peaks with opposite signs at each of the two resonant states (*ε*_*d*_=*μ*,*μ*−*U*) and changes sign whenever *ε*_*d*_ passes each zero points. In the absence of dot-MBSs hybridization (*λ*_1_=0) as indicated by the solid black line in Fig. 2b, *S*_*↑*_ is positive (negative) when *ε*_*d*_ is below (above) the zero point as the main carriers are electrons (holes). With increasing *λ*_1_, the spin-down thermopower *S*_*↓*_ is unchanged and the absolute value of *S*_*↑*_ firstly is suppressed and then enhanced. For sufficiently large *λ*_1_,*S*_*↑*_ changes its sign as shown in Fig. 2b. With further increased *λ*_1_, the absolute value of *S*_*↑*_ exceeds that of *S*_*↓*_ and the total thermopower *S*=*S*_*↑*_+*S*_*↓*_ also changes its sign. Such a phenomenon has also been previously found in the spinless model [35–37]. In fact, the sign change of the thermopower in QD-based device without MBSs was attributed to several causes, such as the system equilibrium temperature [29], magnetic momentum of the electrodes [43], Coulomb interaction [43, 44], coupling strength between the QDs, the applied magnetic field, quantum interference effect, or the magnetic flux penetrating through multiple-dot ones [45, 46]. The above mechanisms are quite different from the present case, and the sign change of the thermopower by changing the hybridization between the QD and the MBSs is helpful for detecting the MBSs [35–37].

**Fig. 3:**
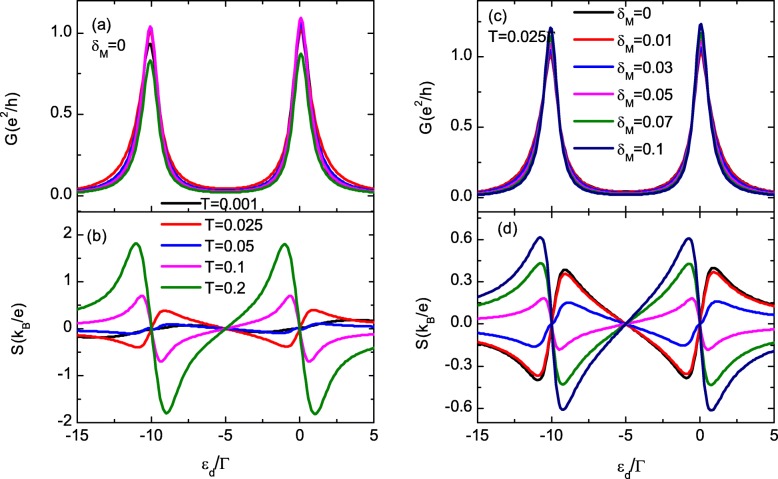
Conductance and thermopower (color online). Counter plot of total conductance *G* and thermopower *S* as functions of *ε*_*d*_ and *Δ*_*M*_ in **a**, **b**, temperature *T* in **c**, **d**, respectively. The value of *λ*_1_ is fixed as 0.2*Γ*. The temperature in **a**, **c** is 0.025*Γ*, and in **c**, **d***Δ*_*M*_=0. Other parameters are the same as those of Fig. 2

Figure 3a, b shows the total conductance *G* and themopower *S* varying with the dot level *ε*_*d*_ for different values of the temperature *T*. The peak value of *G* is firstly enhanced and then suppressed by increasing temperature as shown in Fig. 3a. The magnitude of the thermopower in Fig. 3b, however, is mainly enhanced by increasing temperature, as there are more electrons (holes) excited above (below) the chemical potential. Moreover, *S* changes its sign for the cases of *T*=0.1 and 0.2 as indicated by the pink and green lines in Fig. 3b, which is similar to the case of thermoelectric effect in QD-based structure without MBSs. For *T*=0.2*Γ*, the peak value of *S* can reach as large as 2*k*_*B*_/*e*, which is one order of larger than that of *T*=0.001. In fact, we have checked that the magnitude of the thermopower can be further enhanced by increasing the temperature. In the present paper, however, we focus on the sign change of *S* at relatively low temperature, which is usually the case of the MBSs formed in experiments. Figure 3c, d presents the conductance and the thermopower for different values of direct hybridization of the two MBSs at opposite ends of the nanowire at fixed *T*=0.025*Γ*. The peak value of the conductance in Fig. 3c is monotonously enhanced by increasing *δ*_*M*_, which is in consistent with the results found by López et al. [35]. The thermopower in Fig. 3d changes its sign for 0.03*Γ*<*δ*_*M*_<0.05*Γ*, which is larger than the temperature *T*=0.025*Γ*. In ref. [32], they found that the thermopower changes its sign at about *δ*_*M*_≈*k*_*B*_*T* in the spinless model. In the present paper, the sign change of *S* occurs at relatively larger *δ*_*M*_ as the MBSs are coupled to only one spin direction electrons. Moreover, the peak value of the thermopower can also be enhanced by increasing *δ*_*M*_.

**Fig. 4:**
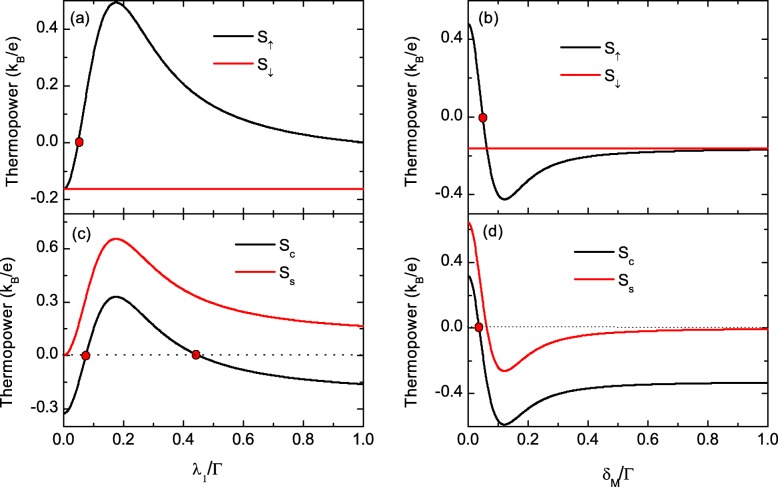
Thermopowers varying with dot-Majorana coupling strength and direct overlap. The thermopowers as functions of *λ*_1_ in **a**, **b** with *Δ*_*M*_=0, and *Δ*_*M*_ in **c**, **d** with *λ*_1_=0.2*Γ*, respectively. Other parameters are the same as those of Fig. 2

We show the spin-resolved thermopowers individually as functions of *λ*_1_ and *δ*_*M*_ in Fig. 4. The spin-up thermopower *S*_*↑*_ in Fig. 4a firstly increases, reaching a maximum and then decreases with increasing *λ*_1_. At sufficiently large *λ*_1_, it remains at a stable value. The value of spin-down thermopower *S*_*↓*_ is unchanged by *λ*_1_ as expected. The behaviors of *S*_*↑*_ and *S*_*↓*_ bring about two interesting results: one is the 100% spin-polarized thermopower when *S*_*↑*_=0 but *S*_*↓*_ has a finite value that can be used for filtering electron spin; the other is the finite pure spin thermopower *S*_*s*_=*S*_*↑*_−*S*_*↓*_ with zero charge thermopower *S*_*c*_=*S*_*↑*_+*S*_*↓*_=0 which occurred when *S*_*↑*_=−*S*_*↓*_ as shown by the dots in Fig. 4b. At closed circuit, the 100% spin-polarized and pure spin thermopowers are individually the corresponding currents, which are virtual in spintronic devices. Similar results are found in Fig. 4b, d, in which *S*_*↑*_ undergoes sign change by changing *δ*_*M*_, whereas *S*_*↓*_ keeps unchanged. We emphasize that the present 100% spin-polarized and pure spin thermopowers emerge in the absence of magnetic field or magnetic materials in the QD.

**Fig. 5:**
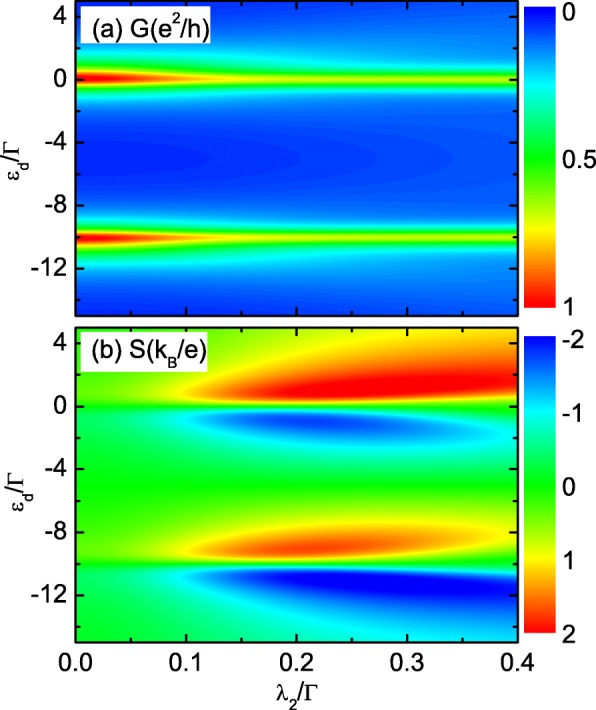
Impacts of the other dot-Majorana coupling on the thermopower (color online). Impacts of *λ*_2_ on the total conductance (**a**) and thermopower (**b**) with *λ*_1_=0.2*Γ*,*δ*_*M*_=0. Other parameters are the same as those of Fig. 2

**Fig. 6:**
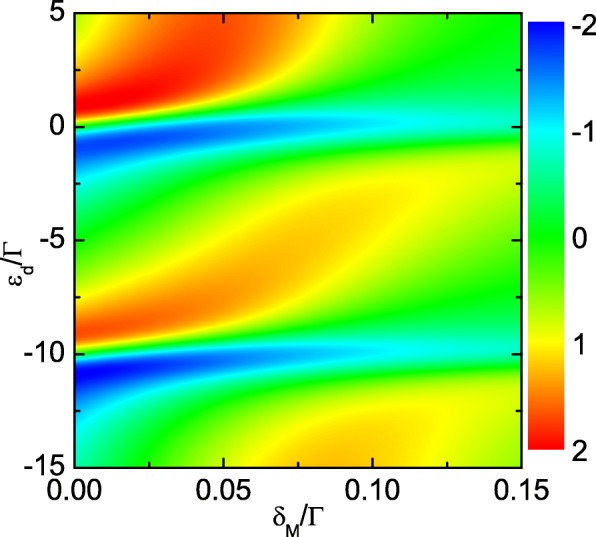
Counter plot of the thermopower (color online). Counter plot of the thermopower as a function of *ε*_*d*_ and *λ*_2_ for *λ*_1_=0.2*Γ*. Other parameters are the same as those of Fig. 2

In Fig. 5, we study the case of both the MBSs at the opposite ends of the nanowire which are coupled to the QD when the wire and the dot are close to each other enough with *δ*_*M*_=0. Figure 5a shows that the total conductance *G* keeps the double-peak configuration in the presence of *λ*_2_. The peaks’ height will be suppressed by increasing *λ*_2_. The lineshape of *S* is also unchanged by the value of *λ*_2_ as indicated by Fig. 5b. The peak’s value of *S* will be significantly enhanced since the thermopower is reversely proportional to the conductance. For *λ*_2_∼0.2*Γ*, the magnitude of the thermopower can reach as large as 2 *k*_*B*_/*e*. Moreover, we find that *S* will not change its sign by adjusting the value of *λ*_2_. Figure 6 shows the total thermopower as a function of *ε*_*d*_ for different values of direct hybridization between the MBSs *δ*_*M*_ by fixing *λ*_1_=*λ*_2_=0.2*Γ*. It shows that both the magnitude and the sign can be effectively changed by tuning *δ*_*M*_, which is similar to the case that only one of the MBSs is coupled to the QD. Finally, we briefly discuss the experimental realization of the present devices. The nanowire hosting the MBSs can be fabricated with InAs grown by molecular beam epitaxy with several nanometers of epitaxial Al layer [47]. It has been experimentally proven that a hard superconducting gap can be induced on such a kind of nanowires [47, 48] by applying a critical magnetic field exceeding 2 T along the wire axis [20]. A QD is formed in the bare InAs segment at the end of the wire due to density of state gradients at the edges of the Al shell [20, 47, 48].

## Conclusions

In conclusion, we have studied the properties of the electrical conductance and thermopower in a quantum dot connected to the left and right normal metal electrodes with Coulomb interaction. The dot is also coupled to MBSs formed in a semiconducting nanowire. We find that the MBSs influence the conductance and thermopower of the spin component it only couples to, although the spin-up and spin-down electrons interact to each other via the Coulomb repulsion. The sign of the thermopower can be changed by adjusting the dot-MBSs hybridization strength, the direction hybridization between the MBSs, and the system temperature. Large value of either 100% spin-polarized or pure spin themropowers can be obtained in non-magnetic QD structure. The coupling between the dot and both the two MBSs can only change the magnitude of the thermopower, but not its sign. Our results may be useful in detecting the existence of the MBSs via thermoelectric technique.

## Data Availability

The datasets supporting the conclusions of this article are included within the article.

## Abbreviations

QD: Quantum dot
MBSs: Majorana bound states
